# The incidence of mental disorder increases after hip fracture in older people: a nationwide cohort study

**DOI:** 10.1186/s12877-021-02195-w

**Published:** 2021-04-15

**Authors:** Ling-Yin Kuo, Po-Ting Hsu, Wen-Tien Wu, Ru-Ping Lee, Jen-Hung Wang, Hao-Wen Chen, Ing-Ho Chen, Tzai-Chiu Yu, Cheng-Huan Peng, Kuan-Lin Liu, Chung-Yi Hsu, Kuang-Ting Yeh

**Affiliations:** 1 School of Medicine, Tzu Chi University, Hualien, Taiwan; 2 Department of Orthopedics, Hualien Tzu Chi Hospital, Buddhist Tzu Chi Medical Foundation, No. 707, ChungYang Rd., Hualien, Taiwan; 3 Institute of Medical Sciences, Tzu Chi University, Hualien, Taiwan; 4 Department of Medical Research, Hualien Tzu Chi Hospital, Buddhist Tzu Chi Medical Foundation, Hualien, Taiwan; 5 Graduate Institute of Clinical Medical Science, China Medical University, Taichung, Taiwan

**Keywords:** Dementia, Depression, Geriatric hip fracture, Nationwide cohort-based study, Transient mental disorder

## Abstract

**Background:**

People living with dementia seem to be more likely to experience delirium following hip fracture. The association between mental disorders (MD) and hip fracture remains controversial. We conducted a nationwide study to examine the prevalence of MD in geriatric patients with hip fractures undergoing surgery and conducted a related risk factor analysis.

**Material and methods:**

This retrospective cohort study used data from Taiwan’s National Health Insurance Research Database between 2000 and 2012 and focused on people who were older than 60 years. Patients with hip fracture undergoing surgical intervention and without hip fracture were matched at a ratio of 1:1 for age, sex, comorbidities, and index year. The incidence and hazard ratios of age, sex, and multiple comorbidities related to MD and its subgroups were calculated using Cox proportional hazards regression models.

**Results:**

A total of 1408 patients in the hip fracture group and a total of 1408 patients in the control group (no fracture) were included. The overall incidence of MD for the hip fracture and control groups per 100 person-years were 0.8 and 0.5, respectively. Among MD, the incidences of transient MD, depression, and dementia were significantly higher in the hip fracture group than in the control group.

**Conclusions:**

The prevalence of newly developed MD, especially transient MD, depression, and dementia, was higher in the geriatric patients with hip fracture undergoing surgery than that in the control group. Prompt and aggressive prevention protocols and persistent follow-up of MD development is highly necessary in this aged society.

## Background

Hip fracture is a common major injury with an increasing incidence in a rapidly aging population. After a severe hip fracture, many active elderly individuals may lose normal physical function [1]. In addition, those with underlying medical problems may no longer be able to live independently, thus leading to increased need of care. At present, with available techniques and surgical procedures, those who fracture a hip are strongly recommended to undergo surgery for pain relief and early mobilization. In the frail population of patients with hip fracture, complications after surgery are inevitable, and high incidence rates of pneumonia (5.9%), surgical site infections (5%), and myocardial infarction (1.9%) have been reported [2]. In addition to physical complications, the most frequent complication during admission after hip fracture surgery is delirium, with incidence rates ranging between 23 and 39% [3–5].

Delirium is an acute change in mental status characterized by fluctuating disturbances of consciousness, attention, cognition, and perception [6]. Elderly patients are vulnerable to developing transient mental disorders (MD), postoperative delirium in particular, due to their chronic comorbid conditions and reduced physiological reserve to address the stress of surgical intervention for hip fracture [7, 8]. In addition, the incidence of dementia is increased in elderly patients with hip fracture, which raises their mortality rates [9, 10]. Delirium is known to increase risk of dementia in the patients aged more than 85 years [11], and those with dementia seem to be more likely to experience delirium following hip fracture [10]. Therefore, the objectives of the present study, which employed a national database, were to examine the association between the older patients with hip fracture undergoing surgical intervention and their risk of postoperative MD.

## Materials and methods

The National Health Research Institutes in Taiwan provided access to the National Health Insurance Research Database (NHIRD) for this study. The NHIRD of Taiwan is a nationwide database covering approximately 99% of Taiwan’s 23.74 million residents who are enrolled in the National Health Insurance (NHI) program, which was launched on March 1, 1995. We used scrambled identifications of residents to link three data collections, namely the Registry of Catastrophic Illnesses Patient Database (RCIPD), Longitudinal Health Insurance Database 2000 (LHID2000), and Registry of Beneficiaries [12]. LHID2000 contains all the original claim data of 200,000 individuals randomly sampled from the 2000 Registry for Beneficiaries of the NHIRD, which maintains the registration data of everyone who was a beneficiary of the NHI program during the period of 1996–2000. There are approximately 23.75 million individuals in this registry. All the registration and claim data of these 1,000,000 individuals collected by the National Health Insurance program constitute the LHID2000. There was no significant difference in the gender distribution between the patients in the LHID2000 and the original NHIRD [13]. The NHIRD records data on diseases on the basis of the *International Classification of Diseases, Ninth Revision, Clinical Modification* (*ICD-9-CM*). This study was approved by the Ethics Review Board of China Medical University and Hospital in Taiwan (CMUH-104-REC2–115).

The hip fracture cohort was identified by seeking patients aged ≥60 years with newly diagnosed hip fracture and receiving further surgical intervention (*ICD-9-CM* codes 820.0–820.9 with open reduction and internal fixation code 64029B or hemiarthroplasty 64170B) within the RCIPD from January 1, 2000, to December 31, 2012. We used the date of surgical treatment of hip fracture received as the index dates and excluded patients with any history of MD before the index date. MD included depression (*ICD-9-CM* codes 296.2, 296.3, 296.82, 300.4, 309.0, 309.1, 311), transient MD (*ICD-9-CM* codes 293, 297), persistent MD (*ICD-9-CM* codes 294.0, 294.8, 294.9), and dementias (*ICD-9-CM* codes 290,294.1, 294.2). The lookback period was 1 year and the same lookback period were applied to all the individuals. The non-hip-fracture cohort comprised subjects without hip fracture during the period 2000–2012 identified from the LHID2000, and the exclusion criteria were the same as for the hip fracture cohort. The non-hip-fracture (control) cohort was frequency matched with the hip fracture cohort at a 1:1 ratio for age, sex, and index year.

All individuals were followed from the index date to the occurrence of MD, death, withdrawal from the NHI program, or December 31, 2012, whichever came first. Some demographic factors and comorbidities that may be associated with MD were also identified. These were sex, age, and comorbidities, including hypertension (HTN; *ICD-9-CM* codes 401–405), diabetes mellitus (DM; *ICD-9-CM* code 250), hyperlipidemia (*ICD-9-CM* code 272), coronary artery disease (CAD; *ICD-9* codes 410–414), cerebrovascular accident (CVA; *ICD-9* codes 430–438), and chronic liver disease (*ICD-9* code 571); incidence of these was compared between the case and control cohorts.

### Statistical analysis

The standardized mean difference for sex, age, and comorbidity and the means of age and follow-up period were applied for further analysis. The incidence rate was defined as the number of events divided by person-years. Crude hazard ratios, adjusted hazard ratios (aHRs), and 95% confidence intervals (95% CIs) were calculated using a multivariable Cox proportional hazards regression model adjusted for sex, age, and comorbidities. We also performed a multivariate with competing risk analysis because death may be a competing risk for the MD event. The competing risk model estimated the subhazard ratios (SHRs) and 95% CIs to indicate the risk of the individual MD between both the matched cohorts. Statistical analysis was performed using SAS 9.4 software (SAS Institute, Cary, NC, USA). *P* < 0.05 was considered statistically significant.

## Results

A total of 1408 patients were enrolled in both the hip fracture group (diagnosis of hip fracture and receiving surgical intervention) and the control group. No significant differences in age, sex, and comorbidities were evident between the control and hip fracture groups (Table 1). The overall incidence of MD for the hip fracture and control groups per 100 person-years were 0.8 and 0.5, respectively (Table 2). The hip fracture group exhibited an increased risk of MD after adjustment for sex, age, and the comorbidities of HTN, DM, CAD, hyperlipidemia, CVA, and chronic liver disease compared with the control cohort (aHR, 1.62; 95% CI, 1.05–2.49). No significant difference was evident between various ages or between sexes (Table 2). The increased incidence of MD in the hip fracture group was found to be similar in all the stratifications by comorbidity. Among MD, prevalence of transient MD was the most significantly higher in the hip fracture group than in the control group (aHR, 3.79; 95% CI, 1.21–11.8). Prevalence of depression and dementia was also significantly higher in the hip fracture group than in the control group (aHR, 1.58; 95% CI, 1.03–2.43; and 1.66, 95% CI, 1.04–2.65, respectively; Table 3). In competing risk of death sensitivity analysis, those who have hip fracture receiving surgery was still associated with higher risk of the transient MD, depression and dementia than those have no hip fracture in the matched controlled cohort (Adjusted SHR, 3.81; 95% CI, 1.15–10.94; 1.55; 95% CI, 1.03–2.42; and 1.91, 95% CI, 1.07–21.94, respectively; Table 4).

**Table 1:** Baseline characteristics in the study cohorts with hip fracture undergoing surgery and without hip fracture as control group

	hip fracture				Standardized mean difference
	No (*n* = 1408)		Yes *(n* = 1408)		
	*n*	%	*n*	%	
Sex					
Female	860	61.1	884	62.8	0.035
Male	548	38.9	524	37.2	0.035
Age, years					
60–69	133	9.45	147	10.4	0.033
70–79	557	39.5	527	37.4	0.044
80+	689	48.9	699	49.6	0.014
Mean (SD)	79.4	8.09	79.2	8.36	0.018
Comorbidity					
HTN	1008	71.6	1079	76.6	0.115
DM	498	35.4	601	42.7	0.15
Hyperlipidemia	470	33.4	442	31.4	0.043
CAD	703	49.9	732	51.9	0.041
CVA	526	37.4	622	44.2	0.139
Chronic liver disease	351	24.9	272	19.3	0.135
Mean of follow-up period of mental disorders (SD)	5.49	3.45	5.16	3.35	0.096

Standardized mean difference ≤ 0.1 indicates a negligible difference between the two cohorts

*Abbreviations: HTN* Hypertension, *DM* Diabetes mellitus, *CAD* Coronary artery disease, *CVA* Cerebrovascular accident

**Table 2:** Incidences and hazard ratios of mental disorder for the hip fracture undergoing surgery cohort and the control cohorts

	Non-hip fracture			Hip fracture			Hazard ratio (95% confidence interval)	
	Event	PY	IR	Event	PY	IR	Crude	Adjusted
Overall	35	7733	0.5	59	7262	0.8	1.71 (1.12–2.59)*	1.62 (1.05–2.49)*
Sex								
Female	26	4702	0.6	41	4802	0.9	1.53 (0.94–2.51)	1.52 (0.92–2.53)
Male	4	3031	0.1	18	2460	0.7	2.12 (0.95–4.72)	2.05 (0.90–4.67)
Age, years								
60–69	5	1006	0.5	13	936	1.4	2.44 (0.87–6.87)	2.50 (0.82–7.61)
70–79	19	3428	0.6	25	3065	0.8	1.39 (0.77–2.53)	1.45 (0.78–2.68)
80+	11	3040	0.4	18	3058	0.6	1.59 (0.75–3.37)	1.62 (0.76–3.46)
Comorbidity								
HTN								
No	9	2580	0.3	13	1904	0.7	1.75 (0.75–4.11)	1.71 (0.72–4.06)
Yes	26	5153	0.5	46	5358	0.9	1.66 (1.03–2.69)*	1.63 (0.99–2.66)
DM								
No	17	5230	0.3	28	4357	0.6	1.88 (1.03–3.45)*	2.08 (1.12–3.84)*
Yes	18	2503	0.7	31	2905	1.1	1.43 (0.80–2.55)	1.42 (0.77–2.61)
Hyperlipidemia								
No	14	5345	0.3	29	5173	0.6	2.02 (1.06–3.82)*	1.84 (0.95–3.54)
Yes	21	2388	0.9	30	2089	1.4	1.55 (0.88–2.71)	1.57 (0.88–2.81)
CAD								
No	12	4042	0.3	23	3663	0.6	2.00 (0.99–4.02)	2.09 (1.02–4.25)*
Yes	23	3691	0.6	36	3599	1	1.52 (0.90–2.57)	1.40 (0.82–2.39)
CVA								
No	18	5272	0.3	25	4281	0.6	1.59 (0.87–2.93)	1.96 (1.06–3.64)*
Yes	17	2461	0.7	34	2981	1.1	1.68 (0.93–3.00)	1.50 (0.82–2.75)
Chronic liver disease								
No	21	5949	0.4	46	6031	0.8	2.05 (1.22–3.44)**	1.98 (1.17–3.35)*
Yes	14	1784	0.8	13	1231	1.1	1.23 (0.58–2.63)	1.08 (0.48–2.41)

Controlling for sex, age, and every comorbidity in Table 2

*Abbreviations: PY* Person-years, *IR* Incidence rate (per 100 person-years), *HTN* Hypertension, *DM* Diabetes mellitus, *CAD* Coronary artery disease, *CVA* Cerebrovascular accident

*P < 0.05, **P < 0.01

**Table 3:** Incidences and hazard ratios of individual outcomes for hip fracture and control cohorts

Outcome	Depression			Hip fracture			Hazard ratio (95% confidence interval)	
	Event	PY	IR	Event	PY	IR	Crude	Adjusted
Depression	35	7733	0.5	58	7271	0.8	1.67 (1.10–2.55)*	1.58 (1.03–2.43)*
Drug-induced mental disorders	1	7931	0.0	1	7576	0.01	1.01 (0.06–16.1)	1.35 (0.08–22.8)
Transient mental disorders	4	7912	0.1	14	7526	0.19	3.70 (1.21–11.2)*	3.79 (1.21–11.8)*
Persistent mental disorder	6	7922	0.1	17	7557	0.22	3.04 (1.19–7.71)*	2.23 (0.85–5.84)
Dementias	31	7885	0.4	48	7468	0.64	1.65 (1.05–2.60)*	1.66 (1.04–2.65)*

Controlling for sex, age, area, every comorbidity and drug in Table 2

*Abbreviations*: *PY* Person-years, *IR* Incidence rate (per 100 person-years)

*P < 0.05

**Table 4:** competing-risk regression of the individual outcomes for hip fracture and control cohorts

Outcome	Hazard ratio (95% confidence interval)	
	Crude SHR	Adjusted SHR
Depression		
Yes	1.62 (1.05–2.51)*	1.55 (1.03–2.42)*
No	1(reference)	1(reference)
Drug-induced mental disorders		
Yes	1.02 (0.05–15.21)	1.32 (0.06–20.43)
No	1(reference)	1(reference)
Transient mental disorders		
Yes	3.65 (1.14–10.81)*	3.81 (1.15–10.94)*
No	1(reference)	1(reference)
Persistent mental disorder		
Yes	2.03 (1.18–22.42)*	1.82 (0.05–14.10)
No	1(reference)	1(reference)
Dementias		
Yes	2.04 (1.19–22.20)*	1.91 (1.07–21.94)*
No	1(reference)	1(reference)

Controlling for sex, age, area, every comorbidity and drug in Table 2

*Abbreviation*: *SHR* Subhazard ratio (per 100 person-years)

*P < 0.05

## Discussion

The recovery of elderly patients who sustain a hip fracture often involves a challenging interaction of physical, psychological, and social factors. Although surgeons have made great advances in surgical techniques and implants, for patients and their families, postoperative care, rehabilitation, and support still impose considerable pressure on their daily lives, economic well-being, and emotions [14]. An estimated 27 to 59% of patients move into permanent long-term care facilities within the first year after a hip fracture [15, 16]. MD, including dementia, depression, and delirium, and geriatric hip fracture often are reciprocal and result in a vicious cycle leading to poor quality of life and death [17–19]. Our nationwide study found that the incidence of MD was significantly higher in the hip fracture group undergoing surgery than in the control group after adjustment for the influence of age, sex, and comorbidities, especially transient MD—mostly delirium, which were 3.79-fold higher in the hip fracture group. A previous meta-analysis revealed that postoperative delirium was related to old age, preexisting cognitive impairment, living in an institution, heart failure, total hip arthroplasty, multiple comorbidities, and morphine use instead of the intraoperative parameters [20, 21]. Patients who experienced an episode of delirium were at increased risk for adverse outcomes [1]. Dementia complicated the elderly a higher hip fx rate, and hip fracture is the third most common cause of admission into an acute setting among the elderly with dementia and leads to high levels of mortality and morbidity [6]. According to strong evidence, an interdisciplinary care program for patients with hip fracture and mild to moderate dementia can improve their functional outcomes [22]. According to a Korean national sample cohort report, patients older than 65 years with depression demonstrated a statistically significant higher hazard ratio for hip fracture than did a control group [23]. Although the outcomes after hip fracture surgery involve various disability levels of mental and physical postoperative pain and severity of complications, the length of hospital stay, psychosocial factors, and symptoms of depression may also increase pain severity and emotional distress. Though some reports have revealed that intraoperative parameters do not significantly correlate with the mental condition of elderly patients [19], others have found that treatment with total hip arthroplasty or hemiarthroplasty for displaced femoral neck fracture, when compared with treatment with internal fixation, appears to achieve better functional outcome [24]. In our study, the hip fracture group had a 1.66-fold higher risk of dementia and 1.58-fold higher risk of depression than the control group after adjustment for other possible risk factors. Subjects we included all were in normal mental status until they fractured a hip; the timeframe within which they developed one of the MD was any time during the postoperative 12 years. Performing routine geriatric mental assessments of hospitalized older patients with hip fracture has been recommended, especially among those older than 81 years and female [1].

The advantage of our study is that the selection and nonresponse biases have been greatly reduced due to the amount of the included people are large with wide-ranging national coverage. We did not include the patients with hip fracture and without receiving surgical intervention in this study because the conservative treatment is not the mainstay treatment strategy for them. The limitations of this study include that the severity of MD, the lifestyle factors, the personal characteristics, and the biochemical data cannot be confirmed and obtained from the NHIRD, which could be the important sources of bias, and these findings based on Taiwanese data may not be directly generalizable to the populations of the other races. Besides, the confirmed diagnosis of MD, such as depression, dementia, delirium or other mental disorders of these patients may be underestimated by using ICD-9-CM to identify them in the database.

Despite these limitations, our paper demonstrates the importance of preventing MD after surgical intervention for hip fractures in the older patients. The vicious chain of dysfunction of mental status and hip fracture found in our study and other studies should be broken so that the quality of life of these older patients can be greatly improved. Through multidisciplinary geriatric rehabilitation [24], physiotherapy interventions [10], and effective prevention of depression [25], physicians, patients, and patients’ family members can cooperate to stop the development and progression of MD.

## Conclusions

Hip fracture affects the physical and mental health of the older individuals greatly. The prevalence of newly developed MD, especially transient MD, depression, and dementia was higher in the geriatric group with hip fracture undergoing surgery than in the control group. Prompt and aggressive prevention protocols and persistent follow-up of MD development appear to be critical in this aged society.

## Data Availability

The datasets used and analyzed during the current study are available from the corresponding author on reasonable request.

## Abbreviations

MD: Mental disorders
NHIRD: National Health Insurance Research Database
NHI: National Health Insurance
RCIPD: Registry of Catastrophic Illnesses Patient Database
LHID: Longitudinal Health Insurance Database
ICD-9-CM: International Classification of Diseases, Ninth Revision, Clinical Modification
HTN: Hypertension
DM: Diabetes mellitus
CAD: Coronary artery disease
CVA: Cerebrovascular accident
aHRs: Adjusted hazard ratios
Cis: Confidence intervals
